# Joint extraction model of entity relations based on decomposition strategy

**DOI:** 10.1038/s41598-024-51559-w

**Published:** 2024-01-20

**Authors:** Ran Li, kaijun La, Jingsheng Lei, Liya Huang, Jing Ouyang, Yu Shu, Shengying Yang

**Affiliations:** 1 Guizhou Power Grid Company Limited, Guiyang, 550000 China; 2 Zhejiang University of Science and Technology, Hangzhou, 310023 China

**Keywords:** Computer science, Information technology

## Abstract

Named entity recognition and relation extraction are two important fundamental tasks in natural language processing. The joint entity-relationship extraction model based on parameter sharing can effectively reduce the impact of cascading errors on model performance by performing joint learning of entities and relationships in a single model, but it still cannot essentially get rid of the influence of pipeline models and suffers from entity information redundancy and inability to recognize overlapping entities. To this end, we propose a joint extraction model based on the decomposition strategy of pointer mechanism is proposed. The joint extraction task is divided into two parts. First, identify the head entity, utilizing the positive gain effect of the head entity on tail entity identification.Then, utilize a hierarchical model to improve the accuracy of the tail entity and relationship identification. Meanwhile, we introduce a pointer model to obtain the joint features of entity boundaries and relationship types to achieve boundary-aware classification. The experimental results show that the model achieves better results on both NYT and WebNLG datasets.

## Introduction

Entity and relationship extraction is a core task in the field of natural language processing for automatically extracting entities and their relationships from unstructured text, and thus constructing relational triples. The results of this task play a crucial role in various advanced natural language processing applications such as knowledge graph construction, question-and-answer systems, and machine translation.

Supervised entity and relation extraction has traditionally used pipelined or joint learning approaches. The pipeline approach views the extraction task as two serial subtasks - named entity identification and relationship classification. The relationship classification subtask pairs the identified entities and then classifies the relationships between the entities. Due to the small number of related entities, the pipeline model usually generates a large number of irrelevant entity pairs in the pairing phase. In addition, the method suffers from error propagation and pays insufficient attention to the relevance of two subtasks. In order to solve these problems, researchers have conducted a lot of research on joint learning and achieved better results.

Joint learning refers to the extraction of entities and classification of relations by a joint model, which can effectively alleviate cascading errors and improve information utilization. Existing studies on joint entity and relation extraction methods focus on the interaction between two subtasks. Zheng et al.^1^ used parameter sharing to make entity identification and relationship extraction interact with each other to achieve the complementary advantages between tasks; Adel and Schütze^2^ used a table-filling approach to fill the diagonal and non-diagonal lines of a table with entity labels and relationship labels, respectively, and model their relationships. Gao et al.^3^ divided extraction into relational extraction and entity recognition. Relationship extraction is transformed into a relationship classification task. Entity recognition is transformed into a sequence marking task. Zhang et al.^4^ proposes a novel relation-specific triple labeling and scoring model, which uses a relational judgment module to predict all potential relationships, prevents computational redundancy, and uses efficient labeling and scoring strategies to decode entities.These methods effectively reduce the impact of cascading errors, but still require entity extraction and relationship classification to be performed independently, and essentially still cannot escape from the pipeline model, while there are a large number of redundant entity pairs^5,6^, because each entity may establish relationships with other entities, and when there are N entities, as many as N2 entity pairs are fed into the relationship classifier, most of which are irrelevant and redundant entity pairs, which affects the accuracy and efficiency of the classifier. At the same time, the above method can not have a more accurate judgment of the entity boundary, especially for the text whose entity boundary information is fuzzy.

In order to solve the above problems, a joint extraction model based on decomposition strategy using pointer mechanism is proposed in this paper. The model divides the joint extraction task into two main parts: head entity recognition and tail entity and relation extraction. First, we apply the header entity recognition technique to identify the main entities in the text. These head entities are then used to provide a gain for tail entity recognition. This hierarchical model design is helpful to improve the accuracy of tail entity recognition. In order to further optimize the performance of the model, we introduce a pointer model to obtain the joint features of the entity boundary and relation type. The pointer model performs well in determining the entity boundary and classifying the relation type, thus realizing the boundary sensing classification. This boundary perception classification method is helpful to better understand and perceive the semantic information and knowledge structure in the text. A large number of experiments on benchmark datasets show that our model has significant advantages and higher performance than previous models. In addition, our model has the advantages of strong interpretability and good robustness. By using the pointer model to obtain the joint characteristics of entity boundary and relation type, we can clearly explain the decision process of the model. This helps us better understand the performance and limitations of the model and provides guidance for future improvements. At the same time, because our model uses a large amount of data and complex algorithms in the training process, it has strong robustness and can adapt to the needs of various natural language processing tasks.

## Related work

Named entity recognition and relation extraction are two important fundamental tasks in natural language processing. The traditional pipeline approach divides this task into two separate subtasks: first extracting named entities from the text, the relationship classification subtask pairing the identified entities, and then classifying the relationships between the entities. While the pipeline-based approach suffers from cascading errors, i.e., the errors in the entity recognition phase are further amplified in the relationship extraction phase, and also fails to facilitate the information interaction between the two subtasks, and also suffers from information redundancy, the joint extraction model can better solve this problem. The joint extraction task is usually solved by a sequence annotation-based approach^7,8^. Miwa and Bansal^9^ used Bi-LSTM to label entities, and then Tree-LSTM was used to resolve and predict the relationships between entity pairs. Bekoulis et al.^10^ formulated it as a head selection problem. Nguyen and Verspoor^11^ apply biaffine attention, Dixit and Al.^12^ use span representation to predict relationships. Miwa and Sasaki^12^ used joint entity and relation extraction as a table filling problem where each cell of the table corresponds to a word pair of the sentence. Gupta et al.^13^ also used joint entity and relation extraction as a table-filling problem, and unlike Miwa and Sasaki, they used a bidirectional recurrent neural network to label each word pair. Lai et al.^14^ introduced an improved graph attention network in the joint model to efficiently extract information from relational nodes. Zhao et al.^15^ proposed a model specific to the relative position representation of entities, which makes full use of the distance information between entities and contextual markers to solve the problems of ambiguous entity features and incomplete local information. Sui et al.^16^ used joint entity and relationship extraction as a direct set prediction problem that can predict all triples at once. Eberts et al.^17^ searches for all spans in the input sentence by strong negative sampling, span filtering, and local contextual representation. Shen et al.^18^ proposed a trigger-aware memory flow framework to enhance the bi-directional interaction between NER and RE tasks through multi-level memory flow attention modules. To address the problem of information redundancy, Zheng et al.^7^ transformed joint extraction into a multi-tagging problem, which makes entity recognition and relation extraction subtasks more coupled and significantly improved compared to the pipeline model, but cannot effectively identify overlapping relations. Dai et al.^5^ proposed a location-attentive long- and short-term neural network model as an improvement, which is based on the location of query words for simultaneous annotation of entities and relations, which achieved better recognition results. However, the model ignores the dependency relationship between head entities and tail entities, which has an important auxiliary value for the recognition of tail entities, and the above model is not conducive to the recognition of overlapping entities and relations.

To sum up, the current named entity recognition and relation extraction tasks mainly use the joint model to eliminate the concatenation error, and we will adopt a similar strategy in our work. At the same time, the above model mainly solves the correlation between the two tasks, but neglects the influence of the head entity on the characteristics of the tail entity, and can not solve the problem of fuzzy entity boundary well.

Inspired by Seo et al.^19^, who achieved machine reading comprehension by predicting the start and end indexes of passages, we designed a joint extraction model using a pointer mechanism decomposition strategy, which uses a pointer mechanism to achieve boundary awareness, identifies head entities first, exploits the positive gain effect of head entities on tail entity recognition, and effectively captures tail entities and relations associated with head entities through a hierarchical model with great success.

## Approach

The main problems solved in this paper are as follows: (1) There are cascade errors in pipeline model; (2) The existing model is not effective in boundary recognition and can not effectively solve the boundary ambiguity problem; (3) Existing models fail to take full advantage of the encoding characteristics between associated entities.

**Figure 1:**
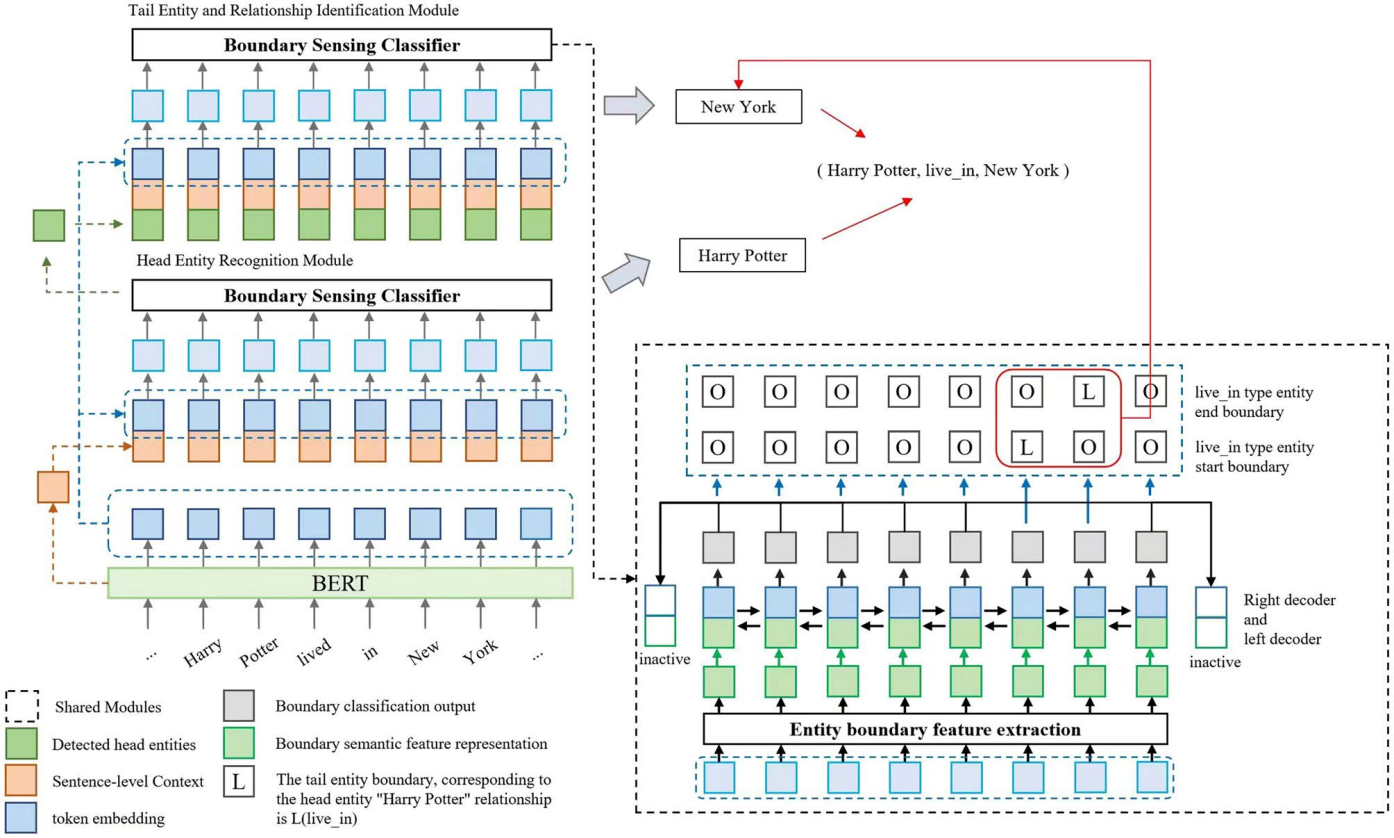
Model structure.The text is input into the BERT model, and the same boundary sensing classifier is used for the head entity and the tail entity. The recognition results of the head entity are sent to the tail entity recognition module to achieve positive gain for the tail entity and relationship recognition, and finally the relationship triplet is obtained.

Our model is a comprehensive framework that consists of four essential components as depicted in Fig. 1. The first component is the BERT[29] encoding layer, which is responsible for converting the input text into a fixed-length vector representation. Bidirectional Encoder Representations from Transformers (BERT) is a pre-trained language model based on Transformer, which can understand the meaning of sentences more accurately through bidirectional training and consideration of contextual information. It uses unsupervised learning to learn language representations by predicting the next word in a sentence, which increases the sensitivity of the model to context.

The second component is the boundary-aware classifier, which is responsible for classifying the input text into specific categories or groups based on its boundary conditions. This component utilizes the encoded output from the BERT encoding layer and applies a series of classifiers to identify the boundary conditions of the input text. The boundary-aware classifier plays a crucial role in ensuring that the input text is correctly classified according to its characteristics.

The third component is the head entity recognition module, which is responsible for identifying the head entity within the input text. This module utilizes the encoded output from the BERT encoding layer and applies a series of algorithms to identify the most relevant entity mentioned in the text that can be considered as the head entity. The head entity recognition module utilizes techniques such as named entity recognition (NER) and information extraction to identify and extract the head entity from the text.

The final component is the tail entity and relationship recognition module, which is responsible for identifying and extracting the tail entities and their relationships within the input text. Similar to the head entity recognition module, this module also utilizes the encoded output from the BERT encoding layer and applies a series of algorithms to extract the tail entities and their relationships mentioned in the text. The tail entity and relationship recognition module utilizes techniques such as NER, information extraction, and relationship extraction to identify and extract the relevant entities and their relationships from the text.

Through the above methods, we can better understand the semantic information and knowledge structure in the text, so as to provide more accurate and comprehensive information for the subsequent tasks.

The encoder of this model is BERT and the output obtained is represented as

$$\begin{aligned} H = B E R T ( S ), \end{aligned}$$

where $S = s _ { 1 }, S _ { 2 } \cdots , S _ { n }$ denotes the text input; $H = h _ { 1 }, h _ { 2 } \cdots , h _ { n }$ denotes the token embedding obtained after each token is encoded by BERT, where $H\in R ^ { n \times d }$, *n* is the number of tokens, *d* is the dimension of the hidden state of BERT, and $h _ { i }$ is the hidden state of position *i*.

### Boundary sensing classifier

In this paper, head and tail entities and relationships are extracted using a unified structure, and this classification method is packaged into a generic module called Boundary sensing classifier (BSC) in this model. To ensure generality, head and tail entities are not distinguished in this subsection, but are referred to collectively as entities.

The probability of extracting an entity target t marked with l from a sentence S is modeled uniformly as

$$\begin{aligned} p ( t, l | S ) = p (s_t^l | S ) p ( e_t^l | s_t^l, S ), \end{aligned}$$

where token *l* denotes the entity type of the head entity or the relationship type of the tail entity, $s_t^l$ is the starting index of *t*, and $e_t^l$ is the ending index. Considering that the prediction of the ending index may be affected by the prediction result of the starting index, this module adopts the structure of hierarchical tokens, associating each layer with a task, and inputting the token results and hidden states of the lower-level tasks to the higher-level tasks.

After obtaining the representation of each word, a pointer mechanism is used for boundary awareness. In this paper, an inactive sentinel word inactive is used to fill the hidden state obtained by the encoder, and the pointer points to the marker inactive if the current input is not an entity boundary. as shown in Fig. 1, there are two types of decoders in the pointer-based network: left decoder and right decoder. Taking the right decoder as an example, an inactive right decoder marker bit is filled in the last position of the hidden state obtained by the encoder, and in the left-to-right decoding, if the input is not the entity boundary, the right decoder pointer is trained to point to the marker bit, and vice versa. That is, the first and last positions of the hidden state h obtained by the encoder are filled with two sentinel vectors, as follows.

$$\begin{aligned} h _ { r } = [ inactive;h;inactive] \end{aligned}$$

where $h'=[h;h_r]$, indicates the hidden state with the starting index fused.

Next, we generate a feature representation for each possible boundary location at time step j. In addition, to provide block-level features, we follow^20^ and add block length information. In summary, the attention mechanism and the length of the data blocks are used to construct the feature representations decoded from left to right as follows.

$$\begin{aligned} u _ { i } ^ { j } = t _ { 1 } ^ { T } \tanh ( W _ { 1 } h _ { i } + M _ { 1 } h _ { r j } ) + t _ { 2 } ^ { T } L E( i - j + 1 ), \quad i \in [ j, n + 2 ] \end{aligned}$$

Similarly, the structure of the feature representation decoded from right to left is as follows.

$$\begin{aligned} u _ { i } ^ { j } = t _ { 3 } ^ { T } \tanh ( W _ { 2 } h' _ { i } + M _ { 2 } h _ { l j } ) + t _ { 4 } ^ { T } L E( i - j + 1 ), \quad i \in [ 0, j ] \end{aligned}$$

Then, the probability that the word $W _ { i }$ is an entity boundary of type is obtained using the Softmax function.

$$\begin{aligned} p ( w _ { i } ^ { t y p e } | w _ { j } ^ { t y p e } ) = {\text {S}} o f t m a x ( u _ { i } ^ { j } ) \end{aligned}$$

In the above equations, $t_1, t_3, t_3, t_4, W_1, W_2, M_1, M_2$ are learnable parameters, $L E ( \cdot )$ is the block length embedding, $i \in [ j, n + 2 ]$ and $i \in [ 0, j ]$ denote the possible positions for left-to-right and right-to-left decoding, respectively, and $p ( w _ { i } ^ { t y p e } | w _ { j } ^ { t y p e } )$ denotes the probability that the entity of a given type type starts (or or end) boundary $w _ { j }$, the probability that word $w _ { i }$ lies at the end (or start) boundary of an entity of type type. When $w _ { j }$ is not a boundary of any entity, the pointer is trained to point to the filled sentinel word. When both $p ( w _ { i } ^ { t y p e } | w _ { j } ^ { t y p e } )$ and $p ( w _ { j } ^ { t y p e } | w _ { i } ^ { t y p e } )$ reach a threshold, this model considers $w = w _ { i, \cdots , } w _ { j }$ is an entity of type type, where this threshold is learned in training and the initial value is 0.5.

During the decoding process, right decoding processes the input in a left-to-right decoding manner, and the index of the ending boundary is always equal to or greater than the index of the starting boundary. When the input word is designated as the start boundary of an entity, the attention probability distribution is calculated and the position of the word that the attention is focused on may vary with each decoding step. Once the end boundaries are determined and the boundary probabilities are all above the threshold, the candidate entity blocks and their types are identified, and the recognition result $\Re _ { B S C }$ is then output, as follows.

$$\begin{aligned} \Re _ { B S C } = \{ w, type _ { w } \} \end{aligned}$$

Boundary Sensing Classifier is used in both modules below.

### Head entity recognition module

This module aims to distinguish candidate head entities and exclude irrelevant head entities. The hidden states $h _ { i }$ and *g* of the encoder are first spliced to obtain the feature vector ${\tilde{s}} _ { i } = [ h _ { i }; g ]$, where *g* is the sentence-level feature representation of the output in BERT. In addition, defining $H _ {head} { } = \{ {\tilde{s}} _ { 1 } , \cdots , {\tilde{s}} _ { n } \}$ denotes all word representations used for head entity recognition, and then $H _ { head }$ is input to a BSC to extract head entities.

$$\begin{aligned} \Re _ { B S C } = B S C ( H _ { h e a d } ), \end{aligned}$$

where $\Re _ { h e a d } = \{ ( e_{j} , t y p e _ { e _{j} } ) \} _ { j = 1 } ^ { m }$ contains all the head entities and corresponding entity type labels in the sentence S.At the same time, the information of the head entity is entered into the following tail entity and relationship recognition module as important auxiliary information.

### Tail entity and relationship identification module

Similar to the head entity recognition module, this module also uses the implicit state $h _ { i }$ of the encoder and sentence-level features *g* as feature inputs. In order to better detect tail entities and their head entity-specific relationships, the key information used in this module are (1) the tail entity’s own features, (2) the corresponding head entity, (3) the contextual feature information, and (4) the head-tail entity spacing. For this purpose, this module uses a feature representation ${\bar{s}}_i$ that incorporates position, context and head entity. ${\bar{s}}_i$ is defined for head entity *e* as follows.

$$\begin{aligned} {\bar{s}} _ { i } = [ h _ { i }; g; h ^ { e }; p _ { i } ^ { e } ], \end{aligned}$$

where $h ^ { e } = [ h _ { s t a r t , } ^ { e } h _ { e n d } ^ { e } ]$ is the representation of the head entity e, $h _ { start } ^ { e }$ and $h _ { e n d } ^ { e }$ are the hidden states of the start and end positions of *e*, respectively, and $p_i^e$ is the position embedding, which encodes the relative distance from $s_i$ to *e*. For a given head entity, there may be more than one tail entity corresponding to it, so after successfully sensing a corresponding tail entity, the model will continue to scan backward and keep searching for the remaining tail entities until the end of the sentence. The model takes $H _ { e n d } = {\bar{s}} _ { 1 } \cdots , {\bar{s}} _ { n }$ as the input of a boundary perceptron BSC.

$$\begin{aligned} \Re _ { e n d } = B S C ( H _ { e n d } ), \end{aligned}$$

where, $\Re _ { e n d } = \{ ( t_j ; , r e l_j ) \} _ { j = 1 } ^ { n }$, $t_j$ denotes the jth extracted tail entity, and $r e l _ { j }$ denotes the relationship label of this entity with the given head entity.

After successfully extracting the head entity e and its corresponding several tail entities, *e* is combined with each $( t _ { j } , r e l _ { j } )$ into a triplet to obtain the final extraction result $\Re$.

$$\begin{aligned} \Re = \{ ( e, r e l_j, t _j ) \} _ { j = 1 } ^ { Z }. \end{aligned}$$

$\Re$ contains all the triples in the sentence S whose head entities are *e*. When model training is performed, *e* is the head entity in the dataset, while when inference is performed, the model selects the head entities from $\Re _{head}$ in turn for the prediction of tail entities and relations.

### Loss function of the model

The model in this paper consists of two subtasks: (1) head entity extraction, and (2) tail entity and relationship extraction. The model uses a weighted loss approach. The weights of this model are denoted as *W*. The model observation noise parameters of the two subtasks are noted as $\sigma _ { h }$ and $\sigma _{tr}$, and the loss function of this model is

$$\begin{aligned} L o s s ( W, \sigma _ { h } \sigma _ { t r } ) = \frac{ 1 }{ 2 \sigma _ { h } ^ { 2 } } Loss _ { h } ( W ) + \frac{ 1 }{ 2 \sigma _ { t r } ^ { 2 } } L o s s _ { t r } ( W ) + \log \sigma _ { h } \sigma _ { t r }. \end{aligned}$$

By minimizing the loss of noise parameters $\sigma _h$ and $\sigma _{tr}$, a balance of subtask losses during training is achieved to optimize the overall performance of the model.

## Experiments

### Datasetsl

In this paper, model performance is tested on three benchmark datasets, NYT-single, NYT-multi and WebNLG.

- NYT-single^21^ is a dataset for single-relation extraction tasks extracted from the New York Times newspaper corpus^22^, The dataset was created using a remote-supervision method applied to the training data annotations, which involved automatically generating training pairs using heuristics and distant supervision rules, and manually annotating the test data. The dataset contains 395 manually annotated sentences, most of which involve single triads with only a few overlapping relations. The NYT-single dataset is partitioned into training, development, and test sets, with 330 sentences in the training set, 35 sentences in the development set, and 30 sentences in the test set. The relations in the dataset cover various aspects of daily life, including people, organizations, events, locations, and other entities. The NYT-single dataset is challenging because it involves long-distance dependencies between entities and multiple relations per sentence.
- NYT-multi^23^ is a dataset for overlapping relation extraction tasks extracted from the NYT-single dataset^23^.It is more challenging than NYT-single because it involves multiple relations within the same sentence, rather than just a single relation. This requires the model to be able to identify multiple relationships between entities within a sentence and to handle the complex dependencies between these relations. The dataset covers a wide range of overlapping relations, including both pairwise and collective relations, as well as nested and recursive relations. It also includes both binary and n-ary relations, with multiple relations extracted from the same sentence. The NYT-multi dataset is partitioned into training, development, and test sets, with 56195 sentences in the training set, 5000 sentences in the development set, and 5000 sentences in the test set. The NYT-multi dataset is challenging because it requires models to handle complex sentence structures and multiple relations within the same sentence.
- The WebNLG corpus consists of factual triads describing facts (entities and relationships between entities) and corresponding factual triads in the form of natural language texts. The corpus contains a collection of 7 triples, each with one or more reference texts. Initially, the dataset was used for WebNLG natural language generation challenge tasks, including mapping collections of triples to text, including reference expression generation, aggregation, lexicalization, and sentence segmentation. The corpus was also used for the reverse task of triad extraction. In this paper, we use the dataset processed by Zeng et al.^23^, with 5000 sentences as the training set, 500 sentences as the validation set, and 700 sentences as the test set.

### Evaluation metrics

The model performance evaluation metrics in this paper include Precision, Recall and F1 value. The specific formula is expressed as follows.

$$\begin{aligned} Precision= & {} \frac{ P \cap R }{ P }\\ {\text { Recall }}= & {} \frac{ P \cap R }{ R }\\ F 1= & {} \frac{ 2 * Precision * R e c a l l }{ Precision + Recall }, \end{aligned}$$

where, *P* denotes the set of triads obtained from the model prediction; *R* denotes the set of real existing triads in the dataset; *F*1 value is used for the overall evaluation of precision and recall.

### Experimental parameter setting

The batch size used in the training process is 32. We choose Adam optimizer and perform linear warm-up to speed up the convergence of the model. Additionally, we adopt learning rate decay with a learning rate of 0.001 and a discard rate of 0.3 to ensure the stability and effectiveness of model training. All the parameters are tuned based on the validation set, including the learning rate, batch size, number of training epochs, and so on. Each experiment is repeated five times with different random seeds for data initialization, and the average results are reported as the final performance.

### Baseline models

In this paper, comparative experiments were conducted with the models of.

- MultiDecoder^23^: this model proposes an end-to-end model for sequence learning based on a replication mechanism and the model has a high generalization capability. Also two different decoding strategies are used in decoding: using only one unified decoder or using multiple independent decoders.
- OrderRL^24^: this model is a sequence-to-sequence model with applied reinforcement learning that considers the order of relational fact extraction in sentences and allows free generation of relations.
- MultiHead^25^: this model proposes a joint neural model that allows simultaneous extraction of entities and relations. It uses a CRF layer to model the entity recognition task and treats the relationship extraction task as a multihead selection problem, potentially identifying multiple relationships for each entity.
- PA-LSTM^5^: this model uses a location-attentive long and short-term neural network, by which all entities and their types, as well as all overlapping relations, can be extracted simultaneously.
- GraphRel^26^: this model proposes an end-to-end relationship extraction model that uses a graph convolutional network for joint learning of entities and relationships, which helps in the prediction of overlapping relationships.
- NovelTagging^7^: this model proposes a new tagging scheme for simultaneous annotation of entities and their relations directly, instead of extracting entities and relations separately.
- ETL^27^: this model redivides the joint extraction task into multiple subtasks, and uses different decoders for different subtasks to achieve multiple classifications.
- GraphJoint: In this model, the relationship extraction task is modeled as mapping from relationship to entity, and text features in sentences are extracted by using graph neural network messaging mechanism, and entity features are further extracted by self-attention mechanism and extended gate convolution.
- TriMF(Trigger-Sense Memory Flow Framework): The model constructs a memory module to memorize the class representations learned in the entity recognition and relationship extraction tasks, and on this basis, designs a multi-level memory flow attention mechanism to enhance the two-way interaction between entity recognition and relationship extraction. The model can enhance the relational trigger information in sentences by triggering sensor module without any manual annotation, thus improving the performance of the model and making the model predictions more interpretable.

### Experimental results

It can be seen from Table 1 that the model in this paper has the highest F1 value for the extraction of triads compared with the baseline model, which proves the effectiveness of the model in this paper.

**Table 1:** Results of different models on NYT and WebNLG test sets.

Dataset	Model	Triplet extraction		
		Precision	Recall	F1
NYT-single	MultiHead	51.5	52.8	52.1
	PA-LSTM	49.4	59.1	53.8
	NovelTagging	61.5	41.4	49.5
	ETL	53.8	65.1	59.0
	TriMF	59.5	64.9	62.1
	GraphJoint	61.3	65.0	63.1
	our model	61.0	66.9	63.8
NYT-multi	MultiHead	60.7	58.6	59.6
	GraphRel	63.9	60.0	61.9
	NovelTagging	32.8	30.6	31.7
	MultiDecoder	61.0	56.6	58.7
	OrderRL	77.9	67.2	72.1
	ETL	83.5	71.7	77.2
	TriMF	85.6	72.1	78.3
	GraphJoint	85.5	72.6	78.5
	our model	86.5	73.2	79.3
WebNLG	MultiHead	57.5	54.1	55.7
	GraphRel	44.7	41.1	42.9
	NovelTagging	52.5	19.3	28.3
	MultiDecoder	37.7	36.4	37.1
	OrderRL	63.3	59.9	61.6
	ETL	84.3	82.0	83.1
	TriMF	83.9	82.7	83.3
	GraphJoint	84.1	83.0	83.5
	our model	85.3	83.1	84.2

The model in this paper uses BERT to encode input text. Compared with other encoders such as LSTM and Bi-LSTM used in other models, BERT has strong concurrency capability and can simultaneously and multi-level extract the feature information of words in sentences. This means that BERT can gain a deeper understanding of the role and meaning of each word in a sentence. In addition, since BERT is able to pick up meaning based on the context of the sentence, it can better handle ambiguity and cases where a word has multiple meanings. This context-understanding capability gives BERT a significant advantage in natural language processing tasks.

In terms of data set NYT-single, F1 value of the model in this paper is significantly improved compared with TriMF and GraphJoint. This is due to the introduction of a boundary-aware classifier, which is particularly concerned with the feature processing of physical boundaries. By strengthening the feature processing of the boundary, our model can identify entities more accurately and improve the accuracy of triplet extraction. Compared to TriMF and GraphJoint, our approach has higher performance in entity recognition.

There are a large number of overlapping entities and relationships in the dataset NYT-multi, but the model in this paper can still maintain the optimal performance on the dataset. This is because we have adopted an innovative decomposition strategy to decompose the joint task into two subtasks: head entity extraction and tail entity and relationship joint extraction. This decomposition method effectively solves the problem of overlapping entities and relations, and improves the accuracy and robustness of the model.

In addition, we use the pointer mechanism to identify and classify the boundary, which further improves the accuracy of the model. TriMF, by contrast, uses a single-sequence labeling approach that does not effectively deal with overlapping entities and relationships. However, ETL adopts the way of head-tail entity hierarchy labeling, but there are still shortcomings in boundary processing. Our method solves these problems by improving boundary processing, thus achieving excellent performance on NYT-multi data sets.

## Ablation study

In this paper, ablation experiments are conducted on the NYT-multi test set to analyze the performance of different modules. The relevant experiments evaluate the contribution of different modules to the overall model performance by replacing module components while controlling for other module designs and model parameters that are identical. Three sets of ablation experiments are set up in this paper, which are.

- BERT: indicates the replacement of the BERT encoder with the Bi-LSTM encoder commonly used in the rest of the baseline model.
- BSC: indicates that the pointer mechanism is removed and the common hidden layer via encoder is used for entity feature extraction.
- Loss: indicates that the weighted loss method is replaced by the direct sum of the losses of the two subtasks.

**Table 2:** Results of ablation studies.

Ablation Methods	Precision	Recall	F1
our model	86.5	73.2	79.3
-BERT	78.5	68.1	72.9
-BSC	73.0	62.9	67.6
-Loss	83.9	69.5	76.0

With the results in Table 2, the following conclusions can be drawn.

1. The use of BERT encoder can extract semantic features better compared to Bi-LSTM, which in turn has a gaining effect on the subsequent tasks.
2. The use of boundary-aware classifiers has a significant effect on the performance improvement of the model, which is due to the pointer mechanism for feature extraction of entity boundaries and the efficiency improvement of hierarchical processing.
3. The use of the weighted loss method can improve the model performance to a certain extent, but considering that there is a hierarchical relationship between two subtasks, and the weighted loss is more suitable for the processing of macroscopically juxtaposed subtasks, the improvement brought by the introduction of this method is not significant compared to other modules.

Obviously, the strategy adopted in this paper can effectively improve the semantic analysis ability of entities and relationships and entity boundary recognition ability.

## Conclusion

In this paper, a joint entity and relationship extraction model based on boundary-aware decomposition strategy is presented. By introducing a pointer mechanism, the extraction of boundary features is effectively facilitated, which in turn improves the boundary-awareness and classification capabilities. The decomposition strategy is also adopted to decompose the joint entity-relationship extraction task into two sub-tasks of head entity identification and tail entity and relationship identification, which improves the identification of overlapping entity relationships. In addition, the model adopts BERT encoder and dynamic weighted loss mechanism to further improve the overall performance of the model. The experimental results show that the model in this paper achieves better results on all three datasets compared to the existing models.

The method proposed in this paper can identify and understand the semantic information and knowledge structure in text more accurately, and provide more accurate recognition information for tasks such as information filtering, knowledge graph construction and question answering system.

## Data Availability

The data that support the findings of this study are available from the corresponding author upon reasonable request.
